# Topical application of *Jaungo* in atopic dermatitis patients: study protocol for a randomized, controlled trial

**DOI:** 10.1186/s13063-017-1920-9

**Published:** 2017-04-12

**Authors:** Younghee Yun, Youme Ko, Jin-Hyang Ahn, Bo-Hyoung Jang, Kyuseok Kim, Seong-Gyu Ko, Inhwa Choi

**Affiliations:** 1 Department of Korean Dermatology, Kyung Hee University Hospital at Gangdong, College of Korean Medicine, Kyung Hee University, Seoul, South Korea; 2 Department of Preventive Medicine, Graduate School, Kyung Hee University, Seoul, Republic of Korea; 3 Department of Ophthalmology, Otolaryngology and Dermatology of Korean Medicine, College of Korean Medicine, Kyung Hee University, Seoul, South Korea

**Keywords:** Dermatitis, Atopic, *Jaungo*, Randomized controlled trial, Phytotherapy, Administration, Application, Topical

## Abstract

**Background:**

Atopic dermatitis (AD) is a common pruritic inflammatory skin disease with increasing prevalence. It can manifest with many different clinical phenotypes; however, in its chronic stage, hyperpigmentation, excoriation, lichenification, and dryness are the main symptoms. *Jaungo* comprises two herbs, *Lithospermi* radix and *Angelica gigantis* radix, and three carrier oils, and is an approved herbal ointment for xerosis cutis in Korea. In past studies, we demonstrated that *Jaungo* had anti-inflammatory and antiallergic activity in in vitro and in vivo AD models; however, there are few relevant randomized controlled clinical trials on Jaungo in AD.

**Methods/design:**

A randomized, double-blind, placebo-controlled, single-center, phase IIa clinical trial was designed to investigate the safety, preliminary efficacy, and dose response of *Jaungo* in AD. The study protocol was approved by the Institutional Review Boards of the Kyung Hee University Korean Medicine Hospital (No. KOMCIRB-160617-HR-027) and the Korea Food and Drug Administration (No. 30907). The study aims to enroll 34 AD patients to be randomly distributed among three parallel groups: treatment 1, treatment 2, and the placebo group. Treatment group 1 applies *Jaungo* twice a day, while treatment group 2 applies *Jaungo* and the placebo ointment once a day, separately, and the placebo group applies the placebo ointment twice a day, for a total of 3 weeks each. Participants will be evaluated for eczema before and after the application of the ointments based on several parameters including the Eczema Area and Severity Index, the SCORing of Atopic Dermatitis Index, the Dermatology Life Quality Index, transepidermal water loss, total IgE level, eosinophil count, and IL-17, IL-22, and IFN-γ levels.

**Discussion:**

The trial is currently ongoing and the enrollment of subjects has been initiated. There is an urgent need to develop a drug for the treatment of dry, hyperpigmented, scaly, and thickened skin in chronic-stage AD. This study will determine the efficacy and safety of *Jaungo* in AD, providing evidence for specific AD symptoms treated by *Jaungo*.

**Trial registration:**

Clinical Trials.gov, identifier: NCT02900131. Registered on 2 September 2016. Korea Clinical Research Information Service, identifier: KCT0002060. Registered on 22 July 2016.

**Electronic supplementary material:**

The online version of this article (doi:10.1186/s13063-017-1920-9) contains supplementary material, which is available to authorized users.

## Background

Atopic dermatitis (AD) is a common pruritic inflammatory skin disease with increasing prevalence in industrialized countries. The prevalence of AD is 5–20% worldwide [1]. Altered immunity and reduced barrier function are fundamental to the development of AD [2], and the heterogeneous pathologic abnormalities observed in this patient population may contribute to the different clinical features seen in each patient. In the acute phase, AD manifests as erythema, microvesiculation, exudation, and crusting; however, in the chronic phase, the main symptoms of AD include hyperpigmentation, excoriation, and dryness. Additionally, severe itch can induce lichenification in the chronic stage owing to protracted rubbing of the skin [3].

The most effective therapy for AD includes short-term treatment of flares and a long-term maintenance approach to skin care designed to prevent or minimize flares [4]. Topical anti-inflammatory glucocorticoids (GCs) are standard therapy; however, long-term use of GCs carries the risk of side effects leading to the need for development of a therapeutic nonsteroidal topical agent.

*Jaungo* is an herbal ointment for xerosis cutis, frostbite, miliaria, anal fissures, and rhus dermatitis and approved by the Korea Food and Drug Administration (KFDA). It is composed of two herbs and three carrier oils: *Lithospermi* radix, *Angelica gigantis* radix, sesame seed oil, bees wax, and swine oil. Previous studies have demonstrated the anti-inflammatory and antiallergic activity of *Jaungo* in in vitro and in vivo AD models [5–7]. Additionally, a case series in 1999 reported the effect of *Jaungo* on AD [8], and two more recent studies reported the effects of *Jaungo* on both radiation dermatitis in breast cancer patients and cutaneous leishmaniasis [9, 10]. However, there are few relevant randomized controlled clinical trials on *Jaungo* for the treatment of AD.

The experimental focus of this trial is to evaluate the safety, preliminary efficacy, and dose response of *Jaungo* in patients having mild-to-moderate AD with hyperpigmentation, excoriation, lichenification, and dryness.

## Methods

### Study setting and design overview

**Fig. 1:**
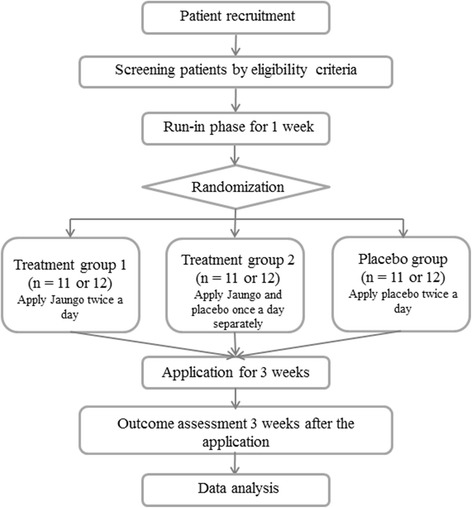
Study flow chart

**Fig. 2:**
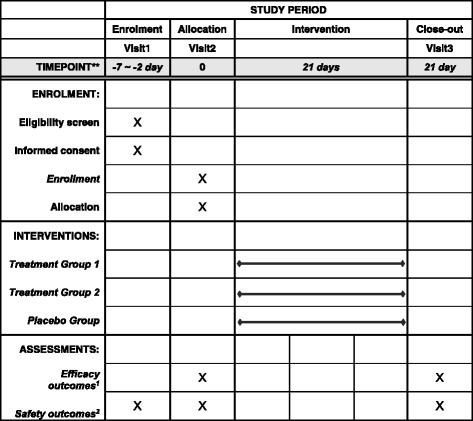
The schedule of enrollment, interventions, and assessments

This study will be conducted at the Kyung Hee University Korean Medicine Hospital in Seoul, Korea. The study is designed as a randomized, double-blind, placebo-controlled, single-center, phase IIa clinical trial to investigate the safety, preliminary efficacy, and dose response of *Jaungo* for treatment of AD. The study aims to enroll 34 patients with AD. Participants fulfilling the eligibility criteria will be selected and randomly distributed into three parallel groups: treatment 1, treatment 2, and the placebo group. Treatment group 1 applies *Jaungo* twice a day; treatment group 2 applies *Jaungo* and placebo ointments once a day, separately; and the placebo group applies placebo ointment twice a day, for a total of 3 weeks each. Participants will be evaluated for AD before and after applying ointment (see Figs. 1 and 2). A completed Standard Protocol Items: Recommendations for Interventional Trials (SPIRIT) Checklist for the trial is available (see Additional file 1).

## Eligible criteria

### Inclusion criteria

Patients will be eligible if they (1) have received a diagnosis of AD according to Hanifin’s and Rajka’s criteria, (2) are men or women aged 5 years to below 65 years, (3) have mild-to-moderate AD symptoms according to the objective SCORing Atopic Dermatitis (SCORAD) Index (score ≤40), and (4) have scores of excoriation, lichenification, and dryness in the SCORAD Index of at least 1 each, or a sum of 3 or more.

### Exclusion criteria

The exclusion criteria are as follows: (1) lesions with oozing, (2) oral administration of corticosteroids, immunosuppressants, or antibiotics 4 weeks prior to study entry, (3) administration of topical GCs, immunosuppressants, or antibiotics, or phototherapy, 2 weeks prior to study entry, (4) burn or trauma on the lesions, (5) allergic to *Jaungo*, or its components, including *Lithospermi* radix, *Angelica gigantis* radix, sesame seed oil, bees wax, and swine oil, (6) active skin diseases without AD, (7) renal or liver dysfunction, (8) other uncontrolled chronic diseases, (9) pregnancy or breastfeeding, (10) participation in other clinical trials within 1 month of enrollment, (11) inability to understand the written consent or to engage in this study due to mental impairment or other emotional or mental problems, and (12) judgment by experts that the potential subject’s participation is inappropriate.

Exclusion will be primarily based on information provided by the patient. Additionally, patients will undergo blood tests for aspartate aminotransferase (AST), alanine aminotransferase (ALT), blood urea nitrogen (BUN), creatinine (Cr), complete blood cell count/differential count (CBC D/C), erythrocyte sedimentation rate (ESR), human chorionic gonadotropin (hCG), and vital signs before the trial to ensure that the patients do not suffer from the listed diseases. A patient with liver dysfunction is defined as having ALT and AST values 2.5 times the upper limit of normal values (over 125 mg/dL for men and over 87.5 mg/dL for women). A patient with renal dysfunction was defined as having a serum Cr value over 2.0 mg/dL.

### Intervention overview

Participants will apply *Jaungo* or placebo ointment for 3 weeks twice a day. The defined daily doses of *Jaungo* should be determined by the KFDA guidelines for specifications and analytical procedures of drug products; however, no pilot study has been performed. Therefore, we are utilizing the previously approved defined daily doses of *Jaungo* for treatment of xerosis cutis. Of note, *Jaungo* is given at a dose twice the strength of topical GCs; however, its anti-inflammatory activity is much less than that of topical GCs.

**Fig. 3:**
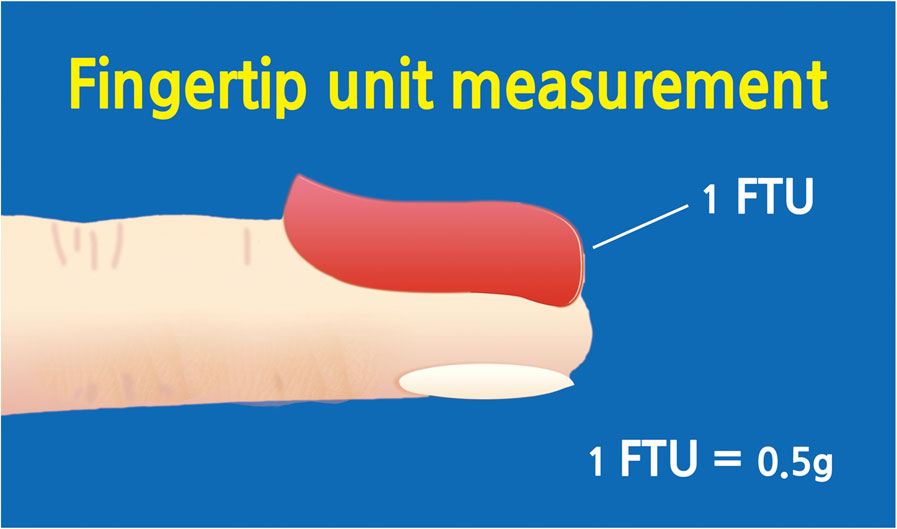
Fingertip unit measurement

The fingertip unit (FTU) measurement will be utilized for participants to determine the required amount of the *Jaungo* to be used (Table 1). One FTU, approximately 0.5 g, is equal to the amount of ointment that is scooped out from the ointment pot with an adult fingertip (Fig. 3). The treatment area for the application of 1 FTU is equal to an area of the skin the size of a flat adult hand with fingers together. Participants will receive instructions for the specific amount of ointment to apply depending on their AD lesions.

**Table 1:** Fingertip unit measurement (FTU) required to cover a specific skin area

Area of skin to be treated	Palm-sized	FTUs each dose (adults)	FTUs each dose (child)
A hand and fingers (front and back)	About 2 palms	2 FTUs (1 g)	1 FTUs (0.5 g)
Front of chest and abdomen	About 14 palms	14 FTUs (7 g)	7 FTUs (3.5 g)
Back and buttocks	About 14 palms	14 FTUs (7 g)	10 FTUs (5 g)
Face and neck	About 5 palms	5 FTUs (2.5 g)	4 FTUs (2 g)
An entire arm and hand	About 8 palms	8 FTUs (4 g)	5 FTUs (2.5 g)
An entire leg and foot	About 16 palms	16 FTUs (8 g)	9 FTUs (4.5 g)

### Experimental medicine (*Jaungo*)

*Jaungo* ointment (Hanpoong Pharm and Foods, Co., Ltd., Wanju, Korea) was produced according to Korea Good Manufacturing Practice standards. *Jaungo* was approved for the treatment of xerosis cutis, frostbite, miliaria, anal fissure, and rhus dermatitis on 11 September 2014, by the KFDA. It is licensed to be applied to skin lesions one or more times per day.

*Jaungo* is composed of two herbs and three carrier oils: 72.7 mg of *Lithospermi* radix, 60.6 mg of *Angelica gigantis* radix, 60.1 mg of sesame seed oil, 242.4 mg of bees wax, and 18.2 mg of swine oil based on a total 1 g of ointment. It is a red-colored ointment with an herbal fragrance. The characteristic red color is due to the *Lithospermi* radix, and the distinctive aroma is due to the flavor components of bees wax and *Angelica gigantis* radix.

### Placebo medicine

Hanpoong Pharm and Foods Co., Ltd., developed a homogenous ointment that is red in color with an herbal fragrance made with 698.1 mg sesame seed oil, 279.2 mg bees wax, 21 mg swine oil, 1.2 mg red iron oxide, and 0.5 mg ginseng-flavored extract based on a total 1 g of ointment. Substances, content, and preparative methods of the placebo ointment were approved by KFDA. The placebo ointment is very similar to the experimental medicine *Jaungo* in color, form, weight, and odor.

### Intervention discontinuation

Participants may discontinue the trial in the following cases: violation of the inclusion/exclusion criteria, violation of combinational medications, the occurrence of serious adverse events, request by participant due to side effects, determination by the investigator due to side effects, systematic disease discovered during the trial, request of participant or their guardian to discontinue, the need for surgery or hospitalization due to accidents or diseases, pregnancy, worsening of atopic dermatitis, continuation judged inappropriate by the investigator.

### Early closure

If participant AD symptoms disappear, the investigator may terminate the case early after discussion with the sponsors and the statistician.

### Adherence assessment

Adherence will be assessed by a container count at visit 3. Participants will bring in experimental medications at visit 3, more than 80% use of experimental medication will be defined as “good adherence,” and less than 80% use will be defined as “poor adherence.” Because the amount of ointment applied by each participant will be different according to the extent of the involved lesion, adherence will be calculated according to the dose of ointment distributed for each participant.

### Combination medications

Different medications for AD, such as orally administered antihistamines, GCs, and immunosuppressants, will not be allowed throughout the study period; however, after discussion with the investigators, patients can self-administer antihistamines orally for controlling pruritus. Patients will also be allowed to use emollients, lotions, and ointments that do not contain GCs during the study. Additional herbal prescriptions, acupuncture treatments, or therapeutic interventions by other clinicians will not be allowed during the study period.

These restrictions are based on the information provided by the patient, and researchers will have to evaluate whether the information provided will affect AD. Drugs taken by each participant will be recorded at every visit, and participants will be asked to notify us of any changes in their medication/supplement regimen. If the participant received medications that may affect AD, they will be excluded from the study.

## Outcomes

### Primary outcome measurement

The primary outcome will be measured by changes in the Eczema Area and Severity Index (EASI) before and after 3 weeks’ application of experimental medications. Key signs of eczema, including erythema (E), infiltration/population (I), excoriation (Ex), and lichenification (L), will be assessed on a numeric scale of 0 to 3. Then, the percentage of area involved for each of the four body regions will be assigned a proportional score from 0 to 6: 0 = no eruption; 1 = <10%; 2 = <10–29%; 3 = <30–49%; 4 = <50–69%; 5 = <70–89%; and 6 = >90–100%. Finally, an established multiplier based on the age of the patient will be used. Please refer to the following formula for patients over 8 years of age. The multiplier for patients under 8 years of age is indicated in parentheses and in italics:

Upper Limbs [(E + I + Ex + L) × Area × 0.2 (*0.2*)] + Lower Limbs [(E + I + Ex + L) × Area × 0.4 (*0.3*)] + Trunk Limbs [(E + I + Ex + L) × Area × 0.3 (*0.3*)] + Head/Neck [(E + I + Ex + L) × Area × 0.1 (*0.2*)] [11].

### Secondary outcome measurements

Secondary outcome measurements will include SCORAD score [12], transepidermal water loss (TEWL) using TM 300 [13], the Dermatology Life Quality Index (DLQI) [14], total IgE, eosinophil count, and interleukin (IL)-17, IL-22, and interferon (IFN)-γ [15]. These parameters will be measured before and after 3 weeks’ application of experimental medications. The consumption of orally administered antihistamines and usage of emollients, lotions, and ointments that do not contain GCs during the study will be checked.

### Safety outcome measurements

Prior to randomization and immediately after treatment completion, we will perform the following tests on all participants: CBC D/C, AST/ALT, BUN/Cr, ESR, and vital signs. These tests will serve to exclude participants who have serious illnesses or abnormal liver, kidney, or other organ function. Additionally, subjective dermal tolerability assessment and the Draize score will be assessed after completion of the study [16].

Listed adverse events verified by the KFDA for *Jaungo* include allergic skin reactions including rash and pruritus. Investigators will assess the incidence and intensity of adverse events. In addition, a causality evaluation of adverse drug reactions will be discussed and documented in Case Report Forms.

### Sample size

This study aims to investigate the efficacy, safety, and dose response for the approved drug, *Jaungo*, with a new indication. *Jaungo* is already approved for use in xerosis cutis, frostbite, miliaria, anal fissures, and rhus dermatitis. There are no previous clinical trials of the use of *Jaungo* in AD. Following the study by Julious [17], we considered the practical feasibility, the possible expectation of the mean and standard deviation, regulations of the KFDA, and risks which fail to detect the difference among the study groups to determine sample size. From this, we decided that 12 patients per group were needed.

### Recruitment

Participants will be recruited by bulletin board advertisements and the online homepages of Kyung Hee University Korean Medicine Hospital, Kyung Hee University Hospital at Gangdong, and Kyung Hee University in Seoul, Korea. Respondents will be contacted by clinical trial coordinators to determine their eligibility via a telephone prescreening. If an applicant meets the study criteria, they will be invited to the clinical research center to be examined for eligibility.

### Randomization and allocation

Before assigning randomization, all participants will be informed that they will be distributed into one of three groups. Thirty-four participants will be randomized into three groups in a 1:1:1 ratio, using block randomization with a block size of 3 or 6, where 11–12 participants are planned for each group. Randomization distribution will occur on the second visit to the clinical research center. Participants will be assigned random numbers via a web-based random number generator system developed by an independent expert on web programming using Django Web Framework (https://www.djangoproject.com). The clinical pharmacy will be supplied with the experimental and placebo ointments in identical containers labeled with random numbers, which correspond to the participant’s assigned number, and “for morning use” or “for evening use.” The participant’s randomly assigned number will dictate which ointments the clinical pharmacist will supply to them.

### Blinding

To ensure blinding, a matched placebo ointment identical in color, texture, weight, and odor will be utilized and stored in a container identical to that of *Jaungo*. The *Jaungo* and placebo ointments will be labeled with a random number and “for morning use” or “for evening use” in an identical form. Treatment group 1 will receive two containers of *Jaungo*, one labeled with “for morning use” and the other “for evening use,” treatment group 2 will receive one container of *Jaungo* and one container of placebo ointment where one of the containers is labeled with “for morning use” and the other “for evening use.” The placebo group will receive two containers of placebo ointment, one labeled with “for morning use” and the other “for evening use.” The clinical pharmacist will supply *Jaungo*, the placebo ointment, or both to the patient depending on the patient’s assigned number. If the ointments are lost or destroyed, the clinical pharmacist will supply the patient with extra ointment labeled in an identical manner to maintain blinding.

In this trial, treatment distribution will remain unknown to the participants, investigators, outcomes assessors, statistician, and other staff until the end of the study. The randomization list and blinding codes will be kept strictly confidential. Access to the randomization list will be limited to the Clinical Research Organization (CRO) staff and the list will only be opened as a standard operating procedure. In the event that the randomization list has to be opened immediately, owing to an urgent situation pertaining to serious adverse events, the primary investigator must report the incident to the CRO. Participants will remain blinded to their respective treatment group until the final visit of the last randomized participant. The success of blinding will be assessed at each participant’s final visit. Researchers who are blinded to the distribution data will perform the outcome assessment. The blinding procedure will also be verified by the authorized CRO.

### Data collection, management, quality control and monitoring

To maintain the quality of this trial, monitoring and data management including data collection, validation, and completion will be conducted by the Institute of Safety and Effectiveness Evaluation for Korean Medicine (ISEE), a CRO located in Seoul, Korea. To ensure that outcome assessments are of a high standard in accordance with the trial protocol, the investigator and the assistants will attend a 6-h training workshop prior to the initiation of the trial. The investigator and the assistants will also be provided with a written protocol and standard operating procedure documents. All the data will be checked regularly by clinical trial coordinators from ISEE.

### Statistical analysis

The primary hypothesis is that topical application of *Jaungo* is more effective than the placebo ointment for treating AD. Baseline data collected on each participant at randomization in the trial will be used to describe the population of patients, assess comparability of treatment groups, achieve balanced randomization, adjust for possible confounding prognostic factors, and undertake subgroup analyses. The baseline characteristics of the three groups, including sex, age, duration of AD, total IgE level, and SCORAD score will be compared. Analysis of variance (ANOVA) will be used to investigate differences in continuously scaled variables, and chi-squared tests will be used to identify significant variations in proportions across treatment groups.

We will also compare the efficacy of *Jaungo* and placebo ointments based on the change in primary outcome, or EASI score, from day 0 to the end of the study period (3 weeks). Mean differences in the EASI score from baseline value to the end of treatment will be compared using ANOVA among the three groups. A paired Student’s *t* test will be used to compare the mean change within a group. Secondary outcomes (SCORAD score, TEWL, DLQI, total IgE, eosinophil count, IL-17, IL-22, and IFN-γ) will also be utilized to compare the efficacy of *Jaungo* and placebo ointments in the same manner as the primary outcome/EASI score. To evaluate safety, the Draize score and adverse events will be presented in a descriptive manner.

Analyses will be performed for two populations: (1) an intention-to-treat population consisting of all randomized participants who have at least one measurable outcome to report following treatment (missing data are replaced with the last observation values) and (2) a per-protocol population including only participants without major protocol deviations. All data will be descriptively analyzed. All main analyses will be based on the intention-to-treat population and conducted using the last observation carried forward imputation method. Statistical analyses will be conducted in a blind manner by an independent statistician and performed using the SPSS 21 (IBM Inc., Armonk, NY, USA), where the level of significance is established at *α* = 0.05.

## Discussion

AD is an eczematous disorder that manifests with erythema, microvesiculation, exudation and crusting in its acute phase, and dry, red, scaly skin in its chronic phase [3]. Treatment guidelines for AD focus on the acute stage of the disease which includes emollients and topical GCs. Topical GCs are the mainstay treatment for AD flare-ups, and in many cases AD can be controlled with topical corticosteroids or topical calcineurin inhibitors; however, some chronic AD patients require other treatment options due to GC side effects.

*Jaungo* is an approved herbal ointment for xerosis cutis, frostbite, miliaria, anal fissures, and rhus dermatitis in Korea. Topical application of *Jaungo* was found to have anti-inflammatory and antiallergic activity in our previous in-vivo and in-vitro studies [5–7]. Based on these findings, we designed a phase IIa clinical study to determine the clinical efficacy of *Jaungo* and its possible treatment mechanism in chronic-stage AD. This preliminary and exploratory study is critical for future confirmatory clinical trials, as no information exists for *Jaungo*’s application dose, frequency, and length of time that it should be administered in AD.

One of the important issues that we had to consider in this study was the blindness between the experimental drug, *Jaungo*, and the placebo ointment. *Jaungo* has a unique color and aroma. The characteristic red color is due to the *Lithospermi* radix, and its distinctive aroma is due to the flavor components of bees wax and *Angelica gigantis* radix. Red iron oxide and ginseng-flavored extract were used to manufacture the placebo ointment. Substances, composition, and manufacturing methods of the placebo ointment were approved by the KFDA. Additionally, to conceal the distribution data from researchers and participants, a web-based random number generator system was developed by an independent expert on web programming using Django Web Framework.

Limitations of this study are that it involves only 34 participants in a single center. This could potentially influence the generalizability of the study results. However, this study was designed to find the most responsive AD symptoms, to establish the proper application dose and frequency of *Jaungo*, and to identify the possible mechanism of action utilized by *Jaungo* for further study. In conclusion, this exploratory study will contribute to establishing *Jaungo*’s efficacy, safety, and dose response in AD.

### Trial status

The trial is currently ongoing. Enrollment of subjects has been initiated.

## Additional file

Additional file 1: SPIRIT Checklist. (DOC 130 kb)

## Abbreviations

AD: Atopic dermatitis
ALT: Alanine aminotransferase
ANOVA: Analysis of variance
AST: Aspartate aminotransferase
BUN: Blood urea nitrogen
CBC D/C: Complete blood cell count differential count
Cr: Creatinine
CRO: Clinical Research Organization
DLQI: Dermatology Life Quality Index
EASI: Eczema Area and Severity Index
ESR: Erythrocyte sedimentation rate
FTU: Fingertip unit
hCG: Human chorionic gonadotropin
ISEE: Institute of Safety and Efficacy Evaluation
KFDA: Korea Food and Drug Administration
SCORAD: SCORing Atopic Dermatitis
TEWL: Transepidermal water loss
